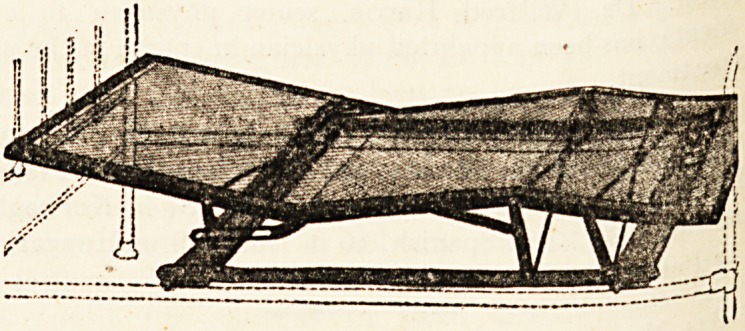# New Appliances and Things Medical

**Published:** 1908-02-15

**Authors:** 


### NEW APPLIANCES AND THINGS MEDICAL.

### THE THOMSON POCKET LAMP.

(L. E. Wilson and Co., 20 Cross Street, Manchester.)

There are many varieties of pocket lamps in which electricity is the illuminant, but we do not think we have as yet seen any variety which surpasses this neat little contrivance. The specimen lamp forwarded for our inspection is a compact apparatus, giving a strong useful light, the value of which, for clinical purposes, is enhanced by the bull's-eye condenser which is fitted to it. A more handy and convenient instrument for illuminating the mouth and throat, for example, it would be difficult to find, and the uses to which such a valuable pocket light may be put by the surgeon are too numerous to specify. The lamp gives a fine steady light for more than four hours, allowing for inevitable leakages, and can be easily recharged by the owner himself at a cost of less than one farthing for electricity by means of the recharging apparatus supplied by the same firm. For dark-room work special yellow and ruby tinted caps are supplied. The price of the lamp is 8s. 6d.

### THE "EQUIPOISE" ADJUSTABLE MATTRESS.

(The Equipoise Couch Co., Ltd., Ashford, Kent.)

This mattress was shown at the Exeter Exhibition, and has lately been on view at the London Medical Exhibition in Vincent Square. At both places it attracted much attention from medical men, and those who have had occasion to use it speak in high terms of its efficiency. It consists of a frame of angle steel, with best wire-woven mattress, locking clutch, brass bearings, brass-domed nuts, and a polished wood base, and it can be folded flat for convenience in transit. The principle of this mattress is the same as that of the "Equipoise" bed, already noticed in these columns—namely, that the invalid is able, by manipulating the special catch, to change his position at will. Almost any position can be adopted, and the physical exertion in manipulating the special catch is so trivial that most patients who will need the mattress will be able to look after their own comfort while on it without needing any assistance from the nurse. The mattress is excellently finished, strong, and well made, and should prove a boon to convalescents.

### LIN AULAGE.

(E'Aulage, St. Etienne, Loire. London Agents: Ernest A. White, 40 Stanhope Gardens, Ilford, Essex.)

This is the newest form of the "modern" linseed poultice, in the shape of a specially prepared plaster, which can be easily and comfortably applied. The linseed, which is free from organic or inorganic impurity, appears to be evenly distributed between several layers of thin gauze impregnated with boric acid. The layer of gauze and seed so arranged is then subjected to pressure and sewn into sheets ready for use. To apply the plaster it is merely necessary to dip a piece of proper size into hot water. The gauze and compressed meal rapidly absorb water, and the remarkable softness and lightness of the preparation make it an excellent poultice. The many uses to which such an antiseptic poultice may be put need hardly be enumerated here.

## Figures and Tables

**Figure f1:**
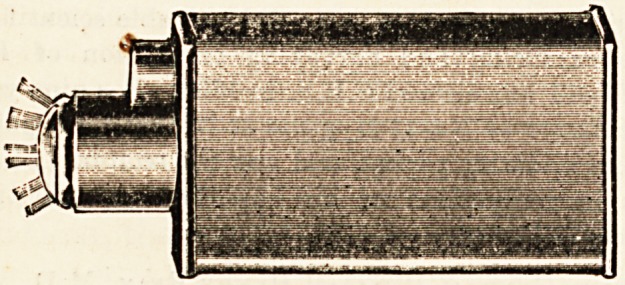


**Figure f2:**